# *pnp4a* Is the Causal Gene of the Medaka Iridophore Mutant *guanineless*

**DOI:** 10.1534/g3.117.040675

**Published:** 2017-03-02

**Authors:** Tetsuaki Kimura, Yusuke Takehana, Kiyoshi Naruse

**Affiliations:** * Division of Human Genetics, Department of Integrated Genetics, National Institute of Genetics, Mishima, Shizuoka 411-8504, Japan; † Laboratory of Bioresources, National Institute for Basic Biology, Okazaki, Aichi 444-8585, Japan; ‡ Department of Basic Biology, School of Life Science, Graduate University for Advanced Studies (SOKENDAI), Okazaki, Aichi 444-8787, Japan

**Keywords:** medaka, iridophore, *pnp4a*, pigment mutant, *guanineless*

## Abstract

See-through medaka lines are suitable for observing internal organs throughout life. They were bred by crossing multiple color mutants. However, some of the causal genes for these mutants have not been identified. The medaka has four pigment cell types: black melanophores, yellow xanthophores, white leucophores, and silvery iridophores. The causal genes of melanophore, xanthophore, and leucophore mutants have been elucidated, but the causal gene for the iridophore mutant remains unknown. Here, we describe the iridophore mutant, *guanineless* (*gu*), which exhibits a strong reduction in visible iridophores throughout its larval to adult stages. The *gu* locus was previously mapped to chromosome 5, but was located near the telomeric region, making it difficult to integrate into the chromosome. We sought the causal gene of *gu* using synteny analysis with the zebrafish genome and found a strong candidate, *purine nucleoside phosphorylase 4a* (*pnp4a*). Gene targeting and complementation testing showed that *pnp4a* is the causal gene of *gu*. This result will allow the establishment of inbred medaka strains or other useful strains with see-through phenotypes without major disruption in the genetic background of each strain.

Medaka embryos are transparent and develop externally, making them suitable for the observation of internal organ developmental processes. However, because of the gradual appearance of pigment cells, observation of internal organs from the early juvenile to adult stages becomes difficult. To solve this problem, the see-through strains ST-II, ST-V, and SK2 were bred by crossing multiple medaka color mutants (Wakamatsu *et al.* 2001; Ohshima *et al.* 2013). In zebrafish, the *casper* fish were made for this purpose. The *casper* fish are doubly mutant for *nacre* (*microphthalmia-associated transcription factor a* mutant) and *roy orbison*. They show a complete lack of melanophores and iridophores and have an almost transparent body from the early juvenile to adult stage (White *et al.* 2008). These see-through strains allow the observation of internal organs throughout life.

To breed transparent medaka strains, multiple crosses among color mutants were necessary. Thus, the original genetic background was disrupted by the crosses. This is undesirable, because uniform and pure genetic backgrounds of inbred lines are one of their advantageous features. Inbred lines are useful for genetic analysis because each inbred line has a unique phenotype (Kimura *et al.* 2007, 2012; Shinya 2011; Tsuboko *et al.* 2014). Thus, to retain the genetic background of inbred lines, another method for creating transparent medaka is required. Recently, clustered regularly interspaced short palindromic repeats (CRISPR)/CRISPR-associated (Cas) system-based RNA-guided endonuclease has emerged as a simple and efficient tool for targeted genome editing in medaka (Ansai and Kinoshita 2014). To apply genome editing for the modification of body color, knowledge of the causal gene for the color mutation is indispensable.

The causal gene of the iridophore-less mutant is the last piece of the jigsaw for making see-through medaka by genome editing. The medaka has four different pigment cells: black melanophores, yellow xanthophores, white leucophores, and silver iridophores. To date, the causal genes of melanophore, xanthophore, and leucophore mutants have been identified (Koga *et al.* 1995; Fukamachi *et al.* 2001, 2004; Kimura *et al.* 2014; Nagao *et al.* 2014), but the causal gene for the iridophore-less mutant remains unknown. Additionally, the causal gene of *roy orbison*, which is responsible for the iridophore-less phenotype in the *casper* fish, is currently unknown. Therefore, our goal was to identify the causal gene for the iridophore-less mutant *guanineless* (*gu*).

The *gu* mutant shows a marked reduction in guanine deposition in iridophores throughout life (Tomita 1992; Kelsh *et al.* 2004) and is a key mutation for the establishment of transparent medaka strains (Wakamatsu *et al.* 2001; Ohshima *et al.* 2013). The *gu* locus has been previously mapped to chromosome 5 (Naruse *et al.* 2000). However, this locus is located near the telomeric region (Naruse *et al.* 1988) and no genome sequence information from around the *gu* locus is available. Thus, the conventional positional cloning approach was inefficient for the *gu* locus. To circumvent this problem, we used the conserved synteny information with the information from zebrafish for gene targeting using the CRISPR/Cas system to identify the causal gene of the *gu* mutant.

## Materials and Methods

### Medaka strains and rearing conditions

Medaka were reared at 26.0° on a 14-hr light/10-hr dark cycle. The T5 strain, which is homozygous for the *gu* mutation, has been described previously (Tomita 1992; Shimada and Shima 2001; Kelsh *et al.* 2004) and used as the *gu* mutant. The d-rR is a closed colony, derived from a southern Japanese population. The d-rR strain was used as wild-type in this study. All the medaka strains were obtained from the National BioResource Project (NBRP), Medaka (www.shigen.nig.ac.jp/medaka/).

### Scaffold mapping

The 95 F_2_ DNA panels from the cross between the Hd-rR and Kaga strains were used for scaffold 1311 mapping. PCR was performed as described previously (Kimura and Naruse 2010). The PCR products were analyzed using a DNA-500 kit on MCE-202 MultiNA (Shmadzu). Scaffold 1311 was mapped by scoring for recombination with the PCR-length polymorphism marker S1311N02 (Supplemental Material, Tables S1 and S2 in File S2).

### Synteny analysis

Synteny analysis was performed using the Genomicus synteny browser (http://www.genomicus.biologie.ens.fr/) (Louis *et al.* 2013, 2015).

### RT-PCR analysis

RNeasy (QIAGEN) and ReverTra Ace (Toyobo) were used to prepare cDNA from embryos. The primers used for amplification are shown in Table S2 in File S2. The PCR products were electrophoresed using a DNA-1000 kit on MCE-202 MultiNA (Shmadzu).

### Genomic PCR analysis

Genomic DNA was extracted from fin clips fixed in 100% methanol. The samples were suspended in 100 ml lysis buffer (10 mM Tris-HCl, pH 8.0; 1 mM EDTA; and 1 mg/ml proteinase K) and incubated at 55° for 3 hr, followed by incubation at 95° for 10 min for proteinase K inactivation. All genomic DNA samples were stored at −20° until use. The PCR conditions for exons 1 and 2–3 were as follows: one cycle at 95° for 2 min; 35 cycles at 95° for 30 sec, 64° for 30 sec, and 72° for 90 sec; followed by 72° for 3 min. The PCR conditions for exons 4–5 and 6–7 were as follows: one cycle at 95° for 2 min; 35 cycles at 95° for 30 sec and 68° for 30 sec; followed by 72° for 1 min. The products were electrophoresed using a DNA-12000 kit on MCE-202 MultiNA (Shmadzu). The primers used for amplification are shown in Table S1 in File S2.

### Gene targeting and mutation identification

Gene targeting using the CRISPR/Cas system was performed as described previously (Ansai and Kinoshita 2014). The sgRNA was designed for exon 2 of *pnp4a* (Figure 3 and Table S1 in File S2), and microinjection was performed using the d-rR strain.

Genomic DNA was purified from fin clips. For sequencing, exon 2 of *pnp4a* was amplified with Ex-taq (TaKaRa). The PCR conditions were as follows: one cycle at 95° for 2 min; 35 cycles at 95° for 30 sec and 68° for 30 sec; followed by 72° for 1 min. The PCR products were treated with ExoSAP-IT (Affymetrix) and sequenced directly. The primers used for amplification and sequencing are shown in Table S1 in File S2.

### Whole-mount in situ hybridization (WISH)

The EST clone, olec1e13, contained the full-length cDNA of *pnp4a*. The partial sequence of the olec1e13 clone was ligated into TOPO-II (Invitrogen) via PCR. The primers used for amplification are shown in Table S1 in File S2. A digoxigenin (DIG)-labeled RNA probe was generated using the DIG RNA labeling kit (Roche). WISH was performed as described previously (Takashima *et al.* 2007).

### Phylogenetic analysis

Phylogenetic analysis was conducted using the neighbor joining method with 2000 bootstrap replicates using MEGA version 7.0.21 (Zuckerkandl and Pauling 1965; Felsenstein 1985; Saitou and Nei 1987; Kumar *et al.* 2016). Amino acid sequences were obtained from GenBank and Ensembl. We excluded the *pnp5b* of *Gasterosteus aculeatus*, because their estimated amino acid sequences were markedly different from those of others.

### Data availability

All medaka strains are available from the NBRP, Medaka (www.shigen.nig.ac.jp/medaka/). The amino acid sequences used in phylogenetic analysis are shown in File S1. Sequence data of medaka Pnp4a, Pnp4b, Pnp5a, and Pnp5b have been deposited at DDBJ/EMBL/GenBank under accession numbers LC177073, LC177074, LC177075, and LC177076, respectively.

## Results

**Figure 1:**
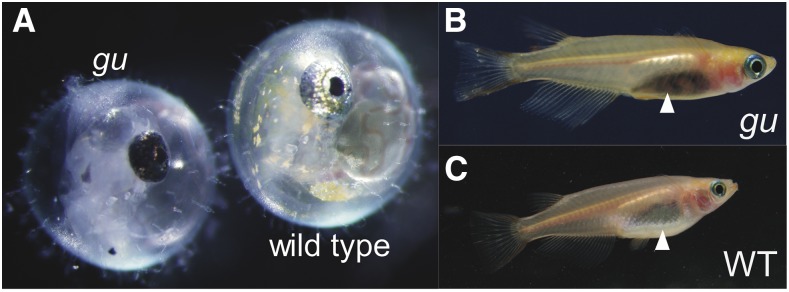
*gu* mutants show a reduction in iridophore pigmentation. (A) The embryo to the left is a 4 dpf *gu* mutant and that to the right is a 4 dpf WT embryo. The eyes of the *gu* mutant appear black compared to those of WT. (B) Photograph of adult fish of the *gu* mutant. The *gu* mutant shows a black abdomen (triangle) because visible iridophores are remarkably reduced. However, the *gu* has silver eyes like those of WT. (C) Photograph of adult fish of WT. The WT has a silver abdomen (triangle). All fish have the *b* mutation. dpf, days postfertilization; *gu*, *guanineless*; WT, wild-type.

The *gu* mutant exhibited drastically reduced guanine deposition in iridophores throughout all life stages (Figure 1) (Tomita 1992; Kelsh *et al.* 2004). The *gu* mutant has a small number of pigmented iridophores. Iridophore differentiation could still proceed normally in the same cells, at least based on the gross appearance of the remaining intact iridophores, but the overall number of iridophores was decreased.

**Figure 2:**
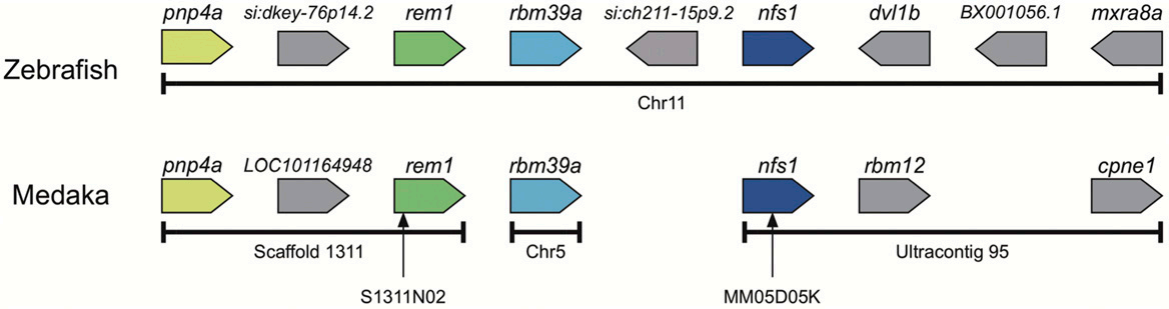
Synteny between medaka and zebrafish suggests that *pnp4a* is near the *gu* locus. The genetic marker MM05D05K is located 0 cM apart from the *gu* locus and is located in *nfs1* on ultracontig 95. Synteny analysis suggests that scaffold 1311 is near ultracontig 95. The polymorphic marker, S1311N02, for mapping the scaffold 1311 position was designed into *rem1*. Pentagons indicate transcriptional orientations. For example, *pnp4a* is transcribed from the left to right. Chr, chromosome; *gu*, *guanineless*; *pnp4a*, *purine nucleoside phosphorylase 4*.

The *gu* locus has already been mapped to chromosome 5 (Naruse *et al.* 2000); however, the *gu* locus is located in the telomeric region of chromosome 5. Unfortunately, the medaka genome sequence lacks this region. Previously, we constructed a linkage map for medaka (Kimura *et al.* 2007). The microsatellite marker, MM05D05K, was the most telomeric side on chromosome 5 in our linkage map. Because MM05D05K was mapped at 0 cM apart from the *gu* locus, we performed a synteny analysis using Genomicus (Louis *et al.* 2013, 2015). In the genome sequence of the scaffold located at the MM05D05K marker, NFS1 cysteine desulfurase (*nfs1*) was on ultracontig 95, which is not assigned to any chromosome. We compared the region surrounding the *nfs1* region in other fish genomes using the Genomicus synteny browser (Louis *et al.* 2015) and found that *pnp4a* was located near *nfs1* on zebrafish chromosome 11 (Figure 2). In addition, medaka chromosome 5 and zebrafish chromosome 11 shared an ancestral chromosome (Kasahara *et al.* 2007). The purine nucleotide phosphorylase metabolizes guanosine to guanine. In particular, *pnp4a* is a marker gene for iridophores in zebrafish (Curran *et al.* 2010). To confirm the result of synteny analysis, we attempted to map the position of *pnp4a*. As *pnp4a* was located on scaffold 1311 in medaka, we created a polymorphic marker named S1311N02 (Figure 2 and Table S1 in File S2) for mapping and determining the position of the scaffold. The mapping panel showed that scaffold 1311 was located on chromosome 5 and was 0 cM apart from the *gu* locus (Table S2 in File S2). Therefore, we thought that *pnp4a* was a strong candidate causal gene on the *gu* locus.

**Figure 3:**
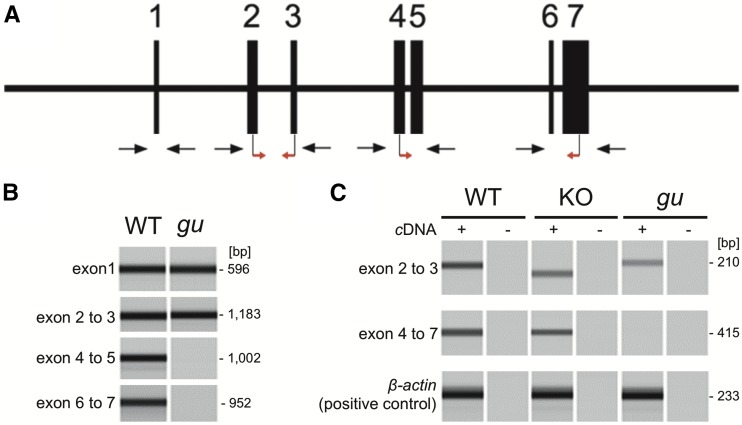
PCR suggests that exon 4–7 of *pnp4a* are lost in the *guanineless* mutant. (A) Scheme of the *pnp4a* gene. The medaka *pnp4a* gene comprises seven exons. Vertical bars indicate exons. Red arrows indicate the positions of primers used in RT-PCR. Black arrows indicate the positions of primers used in genomic PCR. (B) Genomic PCR result of WT and *gu*. Genomic PCR could not detect exon 4–7 in the *gu* mutant. KO indicates *pnp4a* KO. (C) *pnp4a* RT-PCR results of WT, *pnp4a* KO using CRISPR, and the *gu* mutant. RT-PCR could not detect exon 4–7 of *pnp4a* in the *gu* mutant. The amplicon of exon 2–3 of the *pnp4a* knockout is smaller than that of WT and the *gu* mutant because the knockout has a 13-bp deletion in exon 2 induced by CRISPR. β*-actin* is the positive control. cDNA, complementary DNA; Cas, CRISPR-associated; CRISPR, clustered regularly interspaced short palindromic repeats; *gu*, *guanineless*; KO, knockout; PCR, polymerase chain reaction; *pnp4a*, *purine nucleoside phosphorylase 4*; RT-PCR, reverse transcription PCR; WT, wild-type.

In order to determine whether the *gu* mutant possesses a mutation in *pnp4a*, we performed genomic PCR and RT-PCR using genomic DNA as well as total RNA extracted from the wild-type and *gu* embryos. We could amplify exons 1 and 2–3 of *pnp4a*, but could not amplify exons 4–5 and 6–7 when using genomic DNA from the *gu* mutant, whereas all PCR amplifications were positive in the wild-type embryo (Figure 3B). These data suggest that the *gu* mutant lacked exon 4–7 (Figure 3, A and B). We also performed RT-PCR analysis of *pnp4a* mRNA from the *gu* mutant and wild-type. RT-PCR experiments with primers for exon 2–3 were positive both in the *gu* mutant and wild-type, but were negative in the *gu* mutant with primers for exon 4–7. Thus, we concluded that exons 4–7 of *pnp4a* were deleted in the *gu* mutant.

**Figure 4:**
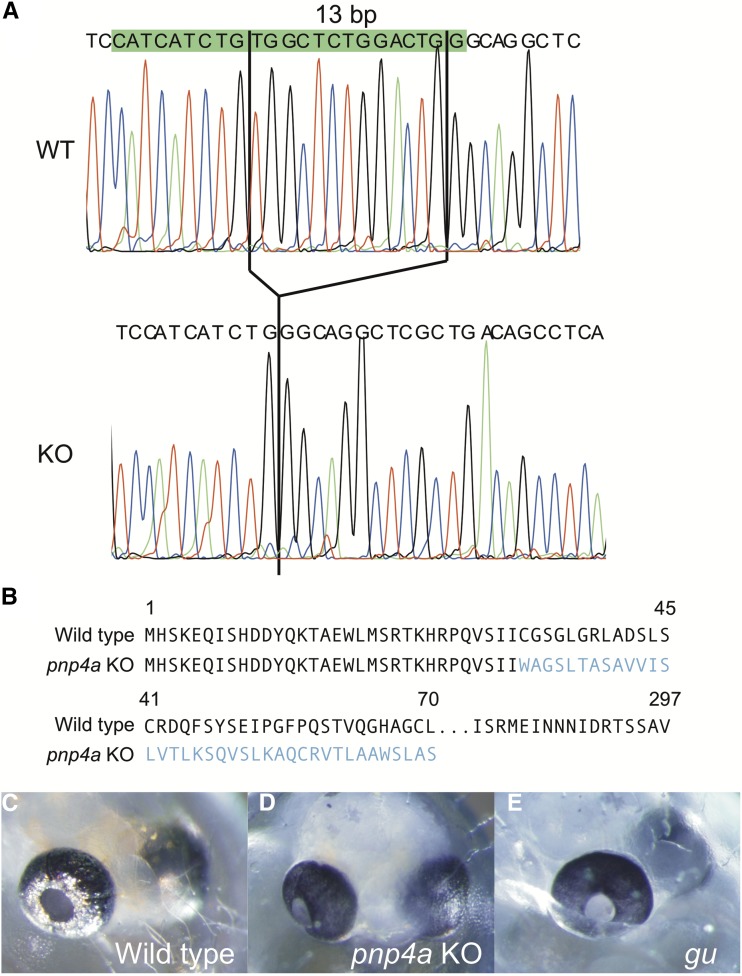
The *pnp4a* gene targeted by the CRISPR/Cas system creates a *gu*-like phenotype embryo. (A) Sequences of the CRISPR/Cas9 target region show a 13-bp deletion in exon 2 of *pnp4a*. The upper panel shows the WT sequence and the lower panel shows the KO fish sequence. Green, blue, black, and red lines indicate the nucleotides A, C, G, and T, respectively. The green box indicates the sgRNA target sequence. (B) Amino acid sequences of *pnp4a* in WT and *pnp4a* KO fish. The 13-bp deletion in exon 2 of *pnp4a* caused a frame-shift and a premature stop in the 71st codon of KO fish. Blue letters indicate the amino acids after the frame-shift. (C) Photograph of the 4 dpf WT embryo. (D) Photograph of the 4 dpf *pnp4a* KO fish (homozygous for the 13-bp deletion). (E) Photograph of the 4 dpf *gu* embryo. The *pnp4a* KO fish has black eyes similar to those of the *gu* mutant. Cas, CRISPR-associated; CRISPR, clustered regularly interspaced short palindromic repeats; dpf, days postfertilization; *gu*, *guanineless*; KO, knockout; *pnp4a*, *purine nucleoside phosphorylase 4*; sgRNA, single guide RNA; WT, wild-type.

**Figure 5:**
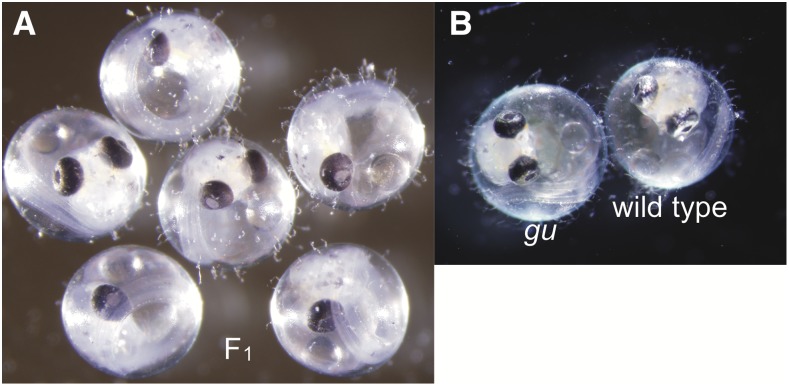
Double heterozygote of the 13-bp deletion and *gu* mutation showed the *gu* phenotype. The 13-bp deletion could not rescue the *gu* phenotype. (A) F_1_ embryos at 5 dpf derived from the cross of the *gu* mutant and the 13-bp deletion homozygote. (B) The *gu* and wild-type embryo at 5 dpf. dpf, days postfertilization; *gu*, *guanineless*.

To confirm that *pnp4a* was the causal gene of the *gu* mutant, we generated *pnp4a* knockout fish using the CRISPR/Cas system (Ansai and Kinoshita 2014). We performed microinjection using one-cell-stage embryos of the d-rR strain. After intercrossing these fish, some progeny exhibited greatly decreased pigmentation of iridophores, a phenotype similar to that of the *gu* mutant. We outcrossed the *gu*-like fish with d-rR and obtained F_1_ fish. As expected, sequence analysis showed that the CRISPR/Cas system induced a 13-bp deletion on exon 2 of *pnp4a* and generated an early stop in the 71st codon of *pnp4a* (Figure 3B and Figure 4, A and B). We then created a homozygote for the 13-bp deletion of *pnp4a*. The *pnp4a* knockout fish exhibited a *guanineless* phenotype indistinguishable from that of the *gu* mutant (Figure 4, C–E). Because the *pnp4a* knockout fish were viable and fertile, we performed a complementation test to confirm that *pnp4a* was the causal gene of the *gu* mutant. We obtained 48 embryos in total from the cross between the *pnp4a* knockout fish (13-bp deletion homozygous) and the *gu* mutant. All embryos showed the same phenotype as that of the *gu* mutants (Figure 5). Thus, we concluded that *pnp4a* was the causal gene of the *gu* mutant.

**Figure 6:**
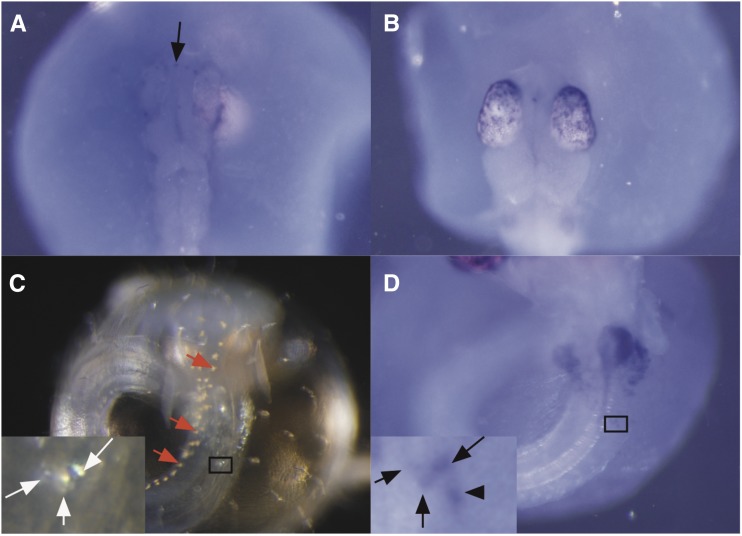
Expression pattern of *pnp4a* at the embryonic stage. (A) *pnp4a* signals were detected as dots (arrow) in the head of 12 somite-stage embryos (2 dpf). (B) *pnp4a* signals were detected in the eyes of 3 dpf embryos. (C) Visible iridophores were observed in 4 dpf embryos. Red arrows indicate leucophores arrayed along the dorsal midline and white arrows indicate iridophores. (D) *pnp4a* signals in the same embryo as that in C. *pnp4a* signals were detected in iridophores (black arrows) but not in leucophores [red arrows in C]. The *pnp4a* signal was also detected in a prepigmented iridophore (triangle). Black squares indicate inset regions in (C and D). dpf, days postfertilization; *pnp4a*, *purine nucleoside phosphorylase 4*.

To determine *pnp4a* expression, we performed WISH. Guanine pigmentation was observed in the eyes of 3-d postfertilization (dpf) embryos, and iridophores emerged on the abdominal region at 4 dpf. Therefore, we used 2, 3, and 4 dpf embryos for WISH. *pnp4a* expression was first detected as dots in the head of 2 dpf embryos (Figure 6A), although there were no visible iridophores in this region. In 3 dpf embryos, the *pnp4a* signal was observed as dots in the eyes (Figure 6B). In 4 dpf embryos, the *pnp4a* signal was observed as pigmented iridophores as well as colorless cells on the abdominal region (Figure 6, C and D), but not in leucophores. These results suggest that *pnp4a* functions as a cell autonomous protein and is the responsible enzyme for iridophore pigmentation.

Since our WISH probe sequence was complementary to exon 4–7 of the *pnp4a* sequence, we performed WISH using *gu* embryos to assess probe specificity. As expected, the 5 dpf embryo of *gu*, which did not possess exon 4–7 of *pnp4a*, did not show any signal (Figure S1A in File S2). Additionally, the 5 dpf *pnp4a* knockout embryo showed signals similar to those in wild-type embryos, consistent with the RT-PCR results (Figure 3C). These results indicate that the probe was highly specific to *pnp4a* mRNA.

**Figure 7:**
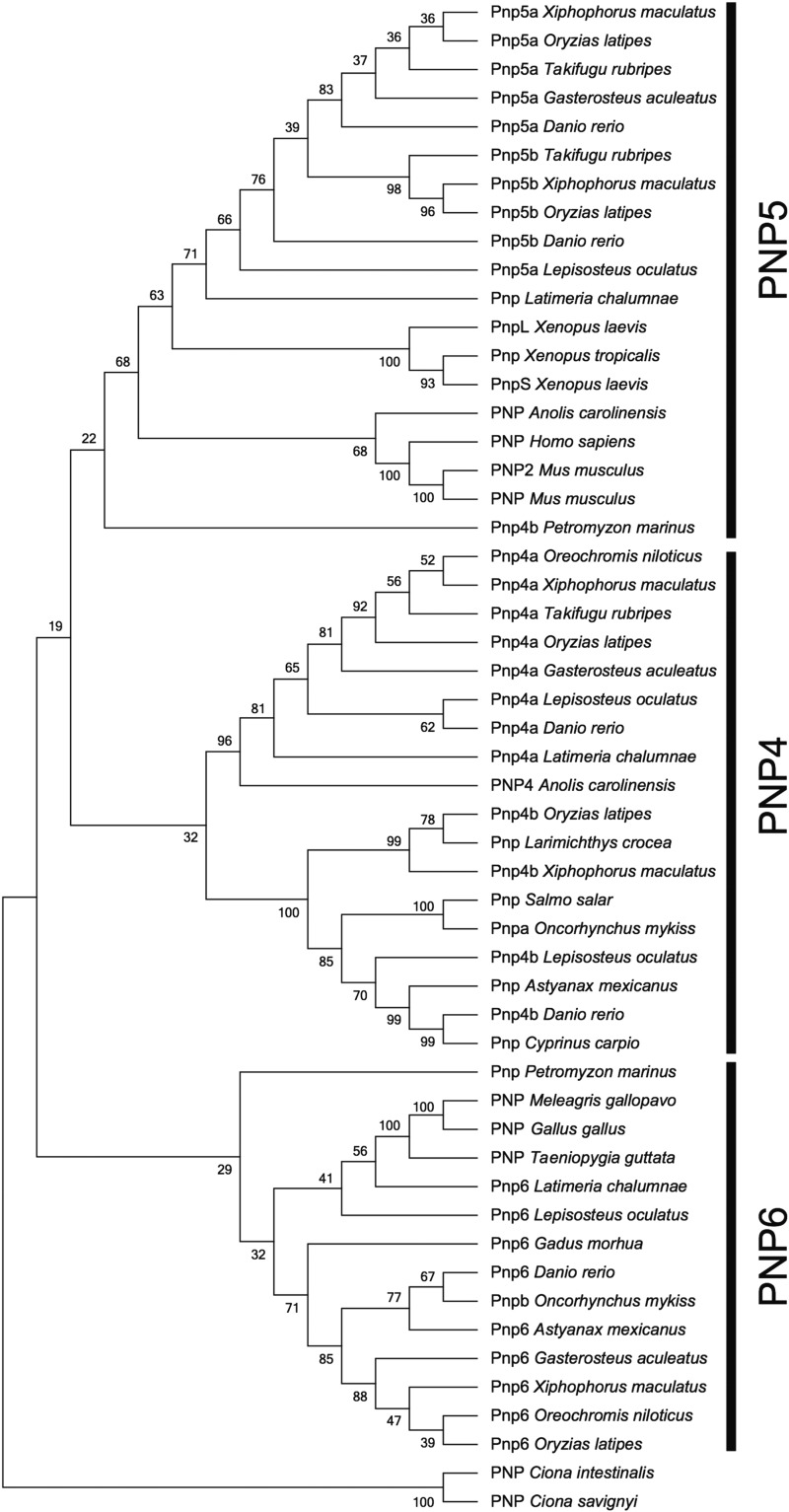
Phylogenetic tree of the *pnp* amino acid sequences. The phylogenetic tree was constructed by Neighbor joining method using MEGA7. Numbers indicate the percentage of replicate trees in which the associated clade clustered together in the bootstrap test (2000 replicates). *pnp*, *purine nucleoside phosphorylase*.

Since *pnp4a* was the causal gene of the *gu* phenotype, we investigated whether *pnp4* has a conserved role in iridophore pigmentation of vertebrates by generating a phylogenetic tree of the *pnp* gene family. The *pnp* gene family of vertebrates is divided into at least three clades, PNP4, PNP5, and PNP6 (Figure 7). Iridophores are conserved among lamprey, teleost, amphibians, and reptiles. On the other hand, *pnp4* genes are conserved in teleost, latimeria, and anole lizards. Although *pnp4a* is conserved in teleosts, *pnp4* gene conservation is not consistent with that of iridophores.

## Discussion

We showed that *pnp4a* was the causal gene of the *gu* mutant and found that *pnp4a*, involving guanine synthesis, was located on the *gu* locus. The *pnp4a* gene was expressed in iridophores in concordance with the *gu* phenotype, which exhibited diminished pigmentation of iridophores. The *pnp4a* knockout experiment showed that *pnp4a* disruption caused a decrease in the pigmentation of iridophores, similar to that in the *gu* mutant. The cross between *gu* and *pnp4a* knockout fish demonstrated that *pnp4a* was the causal gene of the *gu* mutant.

The genomic PCR, RT-PCR, and WISH results indicated that the causal mutation of *gu* was the deletion of exon 4–7 of *pnp4a* (Figure 3, A and B and Figure S1 in File S2). Thus, the *gu* mutation is a null allele of *pnp4a*. The 13-bp deletion in exon 2 of *pnp4a* was also null because the homozygous phenotype was indistinguishable from that of the *gu* mutant (Figure 4). Additionally, *pnp4a* knockout fish expressed mRNA that had the 13-bp deletion, which caused a frame-shift and an early stop codon (Figure 3C).

Although the *gu* mutant has no functional *pnp4a* gene, the *gu* iridophores became slightly pigmented. It is probable that paralogs compensate for *pnp4a* function as another medaka iridophore mutant, *iridophoreless-1* (*il-1*), is reported (Ohshima *et al.* 2013). In the double mutant *gu* and *il-1*, iridophore pigmentation is more repressed than in the single *gu* mutant (Wakamatsu *et al.* 2001; Ohshima *et al.* 2013). This suggests that *pnp4a* is not the only purine nucleoside phosphorylase present in iridophores. In zebrafish, all *pnp* paralogs are expressed in iridophores (Higdon *et al.* 2013). Thus, it is plausible that the causal gene of *il-1* is a purine nucleoside phosphorylase.

Similar to tyrosinase in melanophores, is *pnp4* a common guanine synthetic enzyme in iridophores in vertebrates? Our phylogenetic analysis suggests that it is not (Figure 7). The distribution of *pnp4* is inconsistent with iridophore distribution. Specifically, both *Xenopus* species have iridophores, but they do not have the *pnp4* gene. It is possible that the *pnp4* gene has not yet been discovered in the *Xenopus* genome because *Anolis carolinensis* has *PNP4*. However, birds (*Gallus gallus*, *Meleagris gallopavo*, and *Taeniopygia guttate*) only have the PNP6 gene, suggesting that the subfunctionalization process of *pnp* genes is complicated and that *pnp4* is not a common guanine synthetic gene in vertebrate iridophores. On the other hand, humans and mice with the absence of iridophores only have *PNP5*, whereas *A. carolinensis* with iridophores has *PNP4* and *PNP5*. This suggests that *pnp4* has an important role in guanine synthesis in iridophores. Although further analysis is required to elucidate the relationship between *pnp* genes and iridophore pigmentation, we conclude that *pnp4a* plays a major role in iridophore pigmentation in teleosts.

In this paper, we showed that the *pnp4a* knockout demonstrated an iridophore-less phenotype, and is the first choice to generate an iridophore-less phenotype in fish. Because several causal genes of melanophore mutants are known (Koga *et al.* 1995; Fukamachi *et al.* 2001, 2004) and the *pax7a* mutant has no xanthophores and leucophores (Kimura *et al.* 2014), we expect that our results pave the way for the production of transparent medaka using the CRISPR/Cas system without outcrossing. Since the causal gene of the iridophore-less zebrafish mutant *roy* is not identified, we also hope that our results will be helpful for the establishment see-through strains in zebrafish or in other fish species.

## Supplementary Material

Supplemental material is available online at www.g3journal.org/lookup/suppl/doi:10.1534/g3.117.040675/-/DC1
