# Supplementary material for: pnp4a Is the Causal Gene of the Medaka Iridophore Mutant guanineless
Source: G3 (Bethesda). 2017 Mar 2;7(4):1357–63. doi: 10.1534/g3.117.040675 (PMC5386883; doi:10.1534/g3.117.040675)
Supplement: Supplementary file 2 [file 1357FileS2.docx]

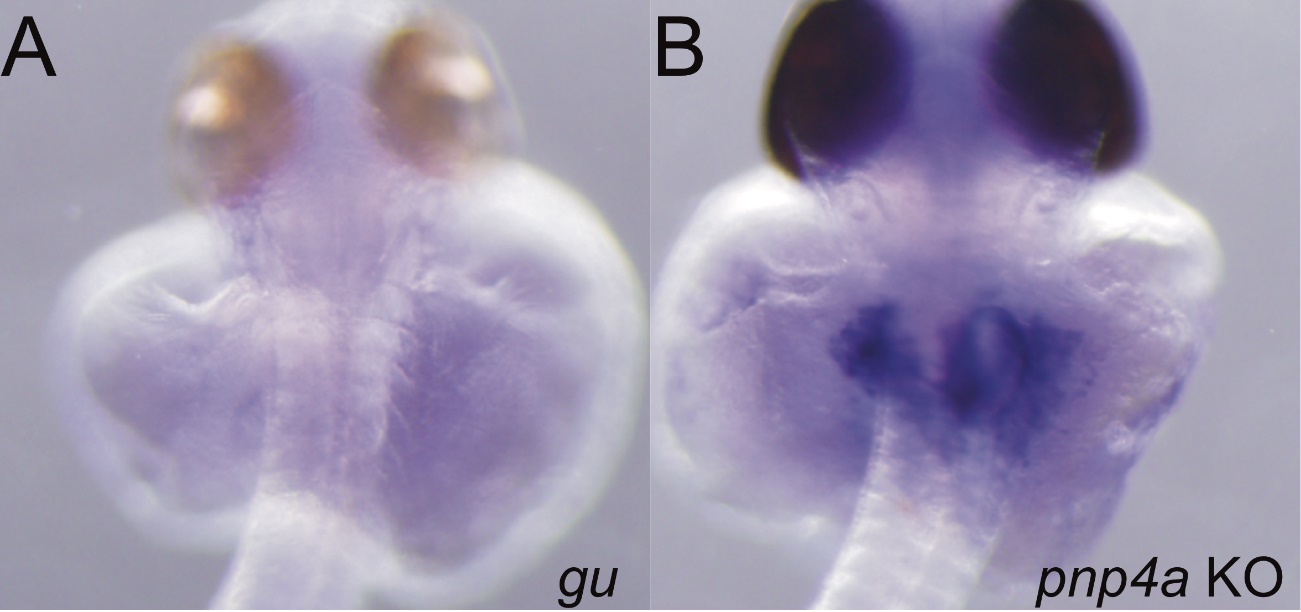


Fig. S1. Expression pattern of *pnp4a* in *gu* and knockout embryos. (A) A *gu* embryo at 5 dpf. (B) A *pnp4a* knockout embryo at 5 dpf. The *pnp4a* signals were detected in *pnp4a* knockout embryos, but not in *gu* embryos. Note the *pnp4a* probe had an anti-sense sequence of exon 4 to 7, which was not detected in the *gu* genome.

Table S1. Primer list.

| **Primer Name** | **Sequence** | **Experiment** |
| --- | --- | --- |
| S1311N02-L | AGATTCTGTAAACTGAAATTGCCCAAATGG | Mapping |
| S1311N02-R | GCAGCTTCTGGTTCTTCACGTTGCTTTGGG | Mapping |
| pnp4a_cDNA-L2 | GCATGCACAGCAAAGAGCAGATCAGCCATG | RT PCR exons 2-3 |
| pnp4a_cDNA-R3 | AAACACACGACCTTCCCTTC | RT PCR exons 2-3 |
| pnp4a_cDNA-L4 | GGTCTGAATCCACTTCATGG | RT PCR exons 4-7 |
| pnp4a_cDNA-R4 | TGCTGATCACTTCTGTCACC | RT PCR exons 4-7 |
| b-actin3b | CMGTCAGGATCTTCATSAGG | RT PCR positive control |
| b-actin4 | CACACCTTCTACAATGAGCTGA | RT PCR positive control |
| Gpnp4aEx1-L | AAAGCTTCACAACAAAAGCCTTTTTGCCTC | Genomic PCR |
| Gpnp4aEx1-R | GGTCTTTTATGGCTCTAACTCTTTTCTTGC | Genomic PCR |
| Gpnp4aEx23-L | AATTGCTGGAATTTTATGACGCCAAATTTG | Genomic PCR |
| Gpnp4aEx23-R | AAGTTCAGATGTTTCAGTCAAATTTCTCCC | Genomic PCR |
| Gpnp4aEx45-L2 | AGTTAAGGCTCCATGCCCTCAGCCTCTGAG | Genomic PCR |
| Gpnp4aEx45-R | TCCCGAAAACACTCACATTCTTACAAACCG | Genomic PCR |
| Gpnp4aEx67-L2 | TCGGACGGATGGATGAAACTGAGAGTGTGG | Genomic PCR |
| Gpnp4aEx67-R2 | GGCCGTGGTGTAGTGGAGACCTGCAGACAC | Genomic PCR |
| pnp4a-oligL | TAGGTCATCTGTGGCTCTGGAC | CRISPR KO |
| pnp4a-oligR | AAACGTCCAGAGCCACAGATGA | CRISPR KO |
| pnp4a_site1-L | CCATGATGACTACCAGAAGACTGCTGAGTG | Sequence |
| pnp4a_site1-R | GATCACGACAGCTGAGGCTGTCAGCGAGCC | Sequence |
| pnp4a-PL | ACTTCCATGTGCAGGACAACGGGGGGGCTG | WISH probe amplify |
| pnp4a-PR | TCTTTAAGCTGATGGGAGTGCAGACGCTGG | WISH probe amplify |

Table S2. Typing results of the polymorphism markers, MM05D05K and S1311N02. In total, 95 F_2_ fish derived from Kaga and Hd-rRII strain crosses were genotyped. Both markers were mapped at the same position. K indicates Kaga homozygous; R, Hd-rRII homozygous; and H, heterozygous alleles.

| Medaka | Marker | |
| --- | --- | --- |
|  | MM05D05K | S1311N02 |
| A01 | H | H |
| A02 | R | R |
| A03 | H | H |
| A04 | H | H |
| A05 | H | H |
| A06 | K | K |
| A07 | K | K |
| A08 | R | R |
| A09 | K | K |
| A10 | K | K |
| A11 | K | K |
| A12 | R | R |
| B01 | R | R |
| B02 | K | K |
| B03 | H | H |
| B04 | H | H |
| B05 | R | R |
| B06 | R | R |
| B07 | R | R |
| B08 | K | K |
| B09 | H | H |
| B10 | R | R |
| B11 | K | K |
| B12 | H | H |
| C01 | H | H |
| C02 | H | H |
| C03 | K | K |
| C04 | H | H |
| C05 | H | H |
| C06 | H | H |
| C07 | K | K |
| C08 | H | H |
| C09 | K | K |
| C10 | H | H |
| C11 | H | H |
| C12 | H | H |
| D01 | R | R |
| D02 | K | K |
| D03 | H | H |
| D04 | H | H |
| D05 | H | H |
| D06 | R | R |
| D07 | R | R |
| D08 | H | H |
| D09 | H | H |
| D10 | H | H |
| D11 | R | R |
| D12 | K | K |
| E01 | K | K |
| E02 | H | H |
| E03 | H | H |
| E04 | H | H |
| E05 | H | H |
| E06 | H | H |
| E07 | R | R |
| E08 | R | R |
| E09 | H | H |
| E10 | K | K |
| E11 | H | H |
| E12 | H | H |
| F01 | H | H |
| F02 | K | K |
| F03 | H | H |
| F04 | H | H |
| F05 | H | H |
| F06 | H | H |
| F07 | R | R |
| F08 | H | H |
| F09 | R | R |
| F10 | K | K |
| F11 | K | K |
| F12 | H | H |
| G01 | H | H |
| G02 | H | H |
| G03 | R | R |
| G04 | H | H |
| G05 | R | R |
| G06 | H | H |
| G07 | H | H |
| G08 | R | R |
| G09 | K | K |
| G10 | R | R |
| G11 | H | H |
| G12 | H | H |
| H01 | H | H |
| H02 | H | H |
| H03 | H | H |
| H04 | K | K |
| H05 | R | R |
| H06 | K | K |
| H07 | R | R |
| H08 | H | H |
| H09 | H | H |
| H10 | K | K |
| H11 | R | R |
